# Chemical screen in zebrafish lateral line identified compounds that ameliorate neomycin-induced ototoxicity by inhibiting ferroptosis pathway

**DOI:** 10.1186/s13578-024-01258-w

**Published:** 2024-06-05

**Authors:** Yipu Fan, Yihan Zhang, Dajiang Qin, Xiaodong Shu

**Affiliations:** 1 Centre for Regenerative Medicine and Health, Hong Kong Institute of Science & Innovation, Chinese Academy of Sciences, Hong Kong SAR, China; 2 Guangdong Provincial Key Laboratory of Stem Cell and Regenerative Medicine, Guangzhou Institutes of Biomedicine and Health, Chinese Academy of Sciences, Guangzhou, 510530 China; 3 University of Chinese Academy of Sciences, Beijing, 100049 China; 4 Bioland Laboratory, Guangzhou Regenerative Medicine and Health Guangdong Laboratory, Guangzhou, 510005 China; 5 Key Laboratory of Biological Targeting Diagnosis, Therapy and Rehabilitation of Guangdong Higher Education Institutes, The Fifth Affiliated Hospital, Guangzhou Medical University, Guangzhou, 510700 China; 6 School of Biological and Chemical Engineering, Zhejiang University of Science and Technology, Hangzhou, 310023 China

**Keywords:** Aminoglycoside, Neomycin, Ototoxicity, Ferroptosis, Zebrafish

## Abstract

**Background:**

Ototoxicity is a major side effect of many broadly used aminoglycoside antibiotics (AGs) and no FDA-approved otoprotective drug is available currently. The zebrafish has recently become a valuable model to investigate AG-induced hair cell toxicity and an expanding list of otoprotective compounds that block the uptake of AGs have been identified from zebrafish-based screening; however, it remains to be established whether inhibiting intracellular cell death pathway(s) constitutes an effective strategy to protect against AG-induced ototoxicity.

**Results:**

We used the zebrafish model as well as in vitro cell-based assays to investigate AG-induced cell death and found that ferroptosis is the dominant type of cell death induced by neomycin. Neomycin stimulates lipid reactive oxygen species (ROS) accumulation through mitochondrial pathway and blocking mitochondrial ferroptosis pathway effectively protects neomycin-induced cell death. We screened an alkaloid natural compound library and identified seven small compounds that protect neomycin-induced ototoxicity by targeting ferroptosis pathway: six of them are radical-trapping agents (RTAs) while the other one (ellipticine) regulates intracellular iron homeostasis, which is essential for the generation of lipid ROS to stimulate ferroptosis.

**Conclusions:**

Our study demonstrates that blocking intracellular ferroptosis pathway is an alternative strategy to ameliorate neomycin-induced ototoxicity and provides multiple hit compounds for further otoprotective drug development.

**Supplementary Information:**

The online version contains supplementary material available at 10.1186/s13578-024-01258-w.

## Introduction

Aminoglycoside antibiotics (AGs, such as neomycin, gentamicin, tobramycin, amikacin, kanamycin, streptomycin, etc.) are broad-spectrum antibiotics that function by irreversibly binding to the 16 S rRNA in the 30 S subunit of bacterial ribosome to disrupt bacterial protein synthesis. Ototoxicity and nephrotoxicity are the two major AG-induced side effects in patients. AGs are transported into hair cells via channels such as mechanotransducer or transient receptor potential where they bind to mitochondrial rRNA and this ribotoxicity is proposed to be critical for AG-induced ototoxicity [1–4]. Mechanistically, AGs induce mitochondrial dysfunction and generate toxic level of intracellular ROS which then leads to hair cell death via multiple intracellular death pathways [4–6]. Based on these discoveries, many drugs or preclinical compounds that can either block AG uptake or scavenge AG-induced ROS have been reported to be otoprotective in cell-based assays or various animal models, however, none of them has been FDA-approved as otoprotective drug so far.

Ferroptosis is a recently discovered form of regulated cell death that is involved in an expanding list of diseased conditions [7–9]. A hallmark of ferroptosis is the accumulation of detrimental level of lipid peroxides which are generated via either Fenton reaction or enzymatic pathways in an iron-dependent manner [10]. Meanwhile, cells have evolved multiple pathways which include the GPX4/GSH pathway, the FSP1/CoQ pathway, the GCH1/BH4 pathway and the mitochondrial DHODH/CoQ pathway to remove excessive lipid peroxides and suppress ferroptosis [8, 11]. Small chemical compounds that inhibit these defensive pathways are able to induce ferroptosis while iron chelators or RTAs inhibit ferroptosis [12]. Several previous studies indicate that lipid ROS and ferroptosis might be involved in AG-induced hair cell damages, for example, the iron chelator deferoxamine partially protects gentamicin-induced hair cell death in cultured guinea pig cochlear neurosensory epithelium [13] and attenuates gentamicin- or neomycin-induced hearing loss in guinea pig in vivo [14, 15]. Sodium selenite which is a GPX4 activator protects against neomycin-induced hair cell damage in zebrafish [16]. The broadly used ferroptosis inhibitor Liproxstatin-1, which is a RTA, reduces neomycin-induced ROS production and cell death in cultured HEI-OC1 cells and cochlear explants [17]. However, direct evidence for a pathogenic role of ferroptosis during AG-induced ototoxicity remains to be established.

AG-induced ototoxicity is traditionally investigated in animal models such as mice, rats, guinea pigs, etc [3]. Recently, zebrafish has become an attractive model for ototoxicity researches [18]. AGs such as neomycin and gentamicin have been established to induce hair cell death in zebrafish inner ear and lateral line [19, 20] and mitochondrial defect has been identified as an early event in neomycin-induced hair cell death in zebrafish [21]. Further study reveals that mitochondrial calcium-driven ROS generation is essential for neomycin-induced hair cell death as mitochondrial-targeted ROS scavenger effectively protects against neomycin ototoxicity [22]. Additional compounds such as trimetazidine, edaravone, Necro-5, ecabet sodium, quercetin are reported to have otoprotective activities against neomycin-induced hair cell death in zebrafish, possibly functioning as RTAs as well [23–27]. In addition to ROS scavenging, blocking the uptake of AGs is another effective way to protect hair cells from AG-induced ototoxicity. For example, berbamine and its derivative compounds, isotetrandrine, d-tubocuraine and ORC-13,661 are reported to protect zebrafish lateral line hair cells from neomycin- or gentamicin-induced ototoxicity by this mechanism [28–31]. Zebrafish is also used as an in vivo screening model to identify additional otoprotective compounds. Screens of several targeted or commercial compound libraries against AG-induced hair cell damage in zebrafish lateral line have been performed and compounds with either previous known or unknown otoprotective activities have been identified [28, 32–37]. Further investigation of these hit compounds will not only shed light on the mechanisms of AG-induced ototoxicity but also provide lead compounds for further otoprotective drug development.

In this study, we report that neomycin induces ferroptosis in zebrafish lateral line hair cells through mitochondrial ROS pathway. Blocking ferroptosis by reducing mitochondrial ROS or lipid ROS prevents AG-induced hair cell damage. We performed a small compound screen and identified additional otoprotective compounds that inhibit ferroptosis and act as either RTAs or regulator of iron homeostasis.

## Results

### Analysis for the relative contributions of apoptosis, necroptosis and ferroptosis pathways in zebrafish hair cell damages induced by various ototoxins

**Fig. 1:**
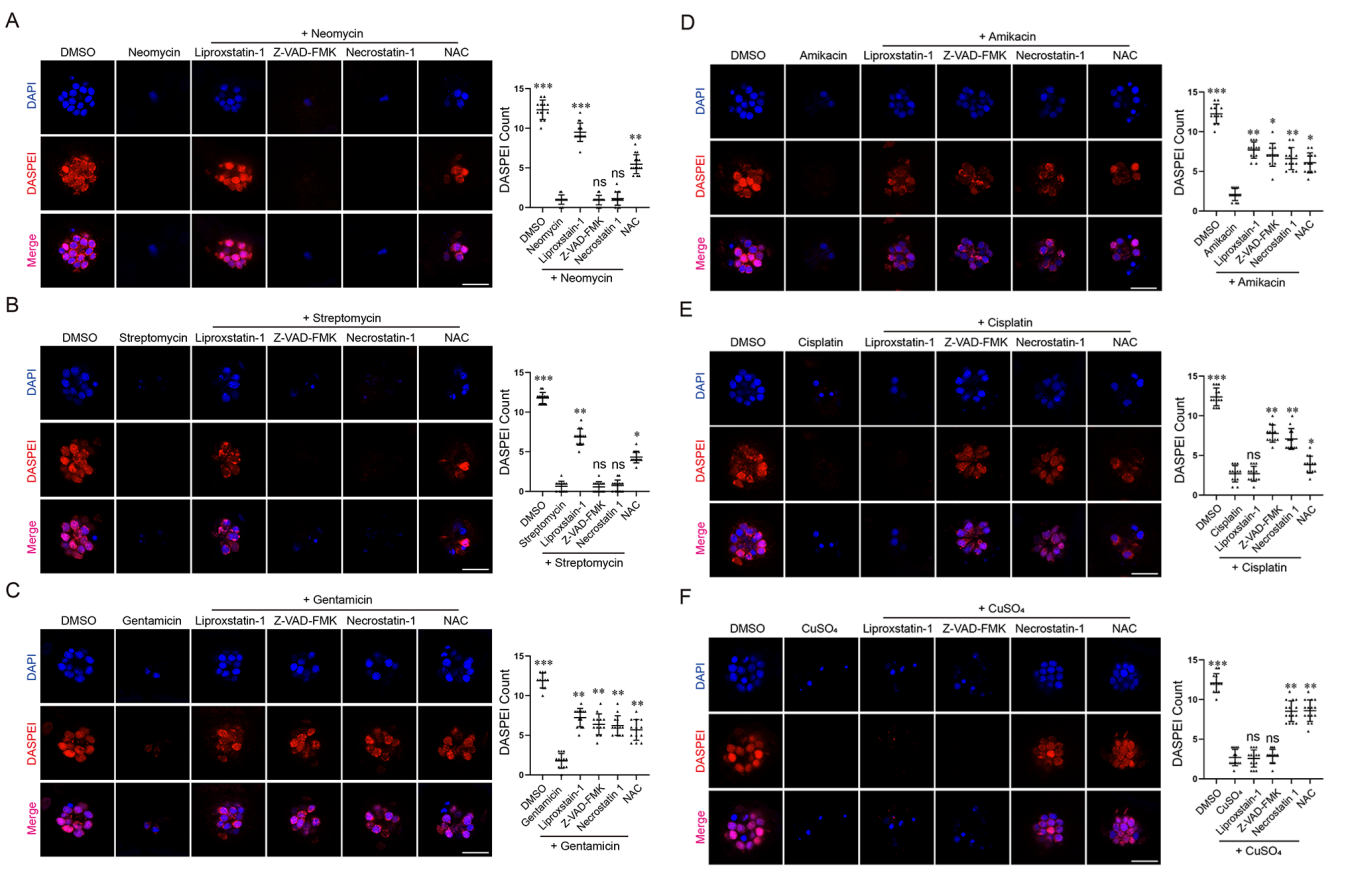
Otoprotection against various ototoxin-induced lateral line neuromast damage by inhibitors of different cell death pathways. Typical confocal images and quantitative results of otoprotection assay. (**A**) The ferroptosis inhibitor Liproxstatin-1 effectively blocks neomycin-induced hair cell death. The non-specific antioxidant NAC shows partial protective activity while apoptosis inhibitor (Z-VAD-FMK) and necroptosis inhibitor (Necrostatin-1) are not effective in this assay. (**B**) Liproxstatin-1 and NAC also partially protect against streptomycin-induced hair cell death. (**C**, **D**) Gentamicin- or amikacin-induced hair cell death can be partially protected by Liproxstatin-1, Z-VAD-FMK, Necrostatin-1, or NAC. (**E**) Cisplatin-induced hair cell damage is ameliorated by Z-VAD-FMK, Necrostatin-1, NAC but not Liproxstatin-1. (**F**) Copper-induce hair cell death is protected by Necrostatin-1 or NAC, but not Liproxstatin-1 or Z-VAD-FMK. Three biological repeats were performed for each assay and at least ten larvae were analyzed for each treatment. Data represent mean ± s.d. and *p*-value is determined by ordinary one-way ANOVA with Dunnett’s multiple comparisons test. ns, no significance; *, *p* < 0.05; **, *p* < 0.01; ***, *p* < 0.001. Scale bar in **A**-**F**: 20 μm

To assess potential contributions of various cell death pathways in AG-induced hair cell toxicity, we first performed otoprotection assay using pathway specific inhibitors. Zebrafish larvae at 5 *day post fertilization* (*dpf*) were pre-incubated with either a ferroptosis inhibitor [38] (Liproxstatin-1, 10 µM), apoptosis inhibitor [39] (Z-VAD-FMK, 50 µM), necroptosis inhibitor [40] (Necrostatin-1, 50 µM) or the general antioxidant N-acetylcysteine which at high concentration ameliorates either necroptosis or ferroptosis (NAC, 5 mM) for 2 h then treated with 125 µM neomycin for 2 h. Treated larvae were stained with 2-[4-(Dimethylamino)styryl]-1-ethylpyridinium iodide (DASPEI, a mitochondrial dye that labels vital hair cells) and 4’,6-diamidino-2-phenylindole (DAPI, which labels hair cell nuclei), imaged under a confocal microscope and the number of vital hair cells per neuromast was counted (at least 10 larvae were assayed in each treatment). A typical result of otoprotection against neomycin-induced hair cell damage is present in Fig. 1A and it reveals that Liproxstatin-1 effectively protects neomycin-induced ototoxicity, NAC has mild protective activity while Z-VAD-FMK or Necrostatin-1 has no detectable protective activity at all. These observations indicate that ferroptosis is the dominant cell death induced by neomycin.

We then analyzed additional AG-induced hair cell damages (streptomycin, gentamicin, and amikacin) using similar protocols and found that streptomycin-induced hair cell death can be protected by Liproxstatin-1 but not Z-VAD-FMK or Necrostatin-1 (Fig. 1B), which is very similar to neomycin-induced hair cell damage. On the other hand, gentamicin- or amikacin-induced hair cell damages can be partially rescued by Liproxstatin-1, Z-VAD-FMK, Necrostatin-1, or NAC (Fig. 1C, D), indicating that ferroptosis, apoptosis and necroptosis all partially contribute to hair cell damages induced by these AGs. We also analyzed cisplatin and CuSO_4_ (two well-established ototoxic reagents in zebrafish) [20, 41] induced hair cell damage and found that cisplatin-induced ototoxicity can be protected by apoptosis and necroptosis inhibitor but not ferroptosis inhibitor (Fig. 1E) while CuSO_4_-induced hair cell death can be blocked by necroptosis inhibitor or NAC but not ferroptosis or apoptosis inhibitor (Fig. 1F). Together, these data indicate that various intracellular cell death pathways are activated by different ototoxic reagents and ferroptosis is the dominant cell death induced by neomycin or streptomycin, it also contributes to gentamicin- or amikacin-induced hair cell death but it is dispensable for cisplatin- or CuSO_4_-induced hair cell death.

### Neomycin induces lipid ROS accumulation in hair cells

**Fig. 2:**
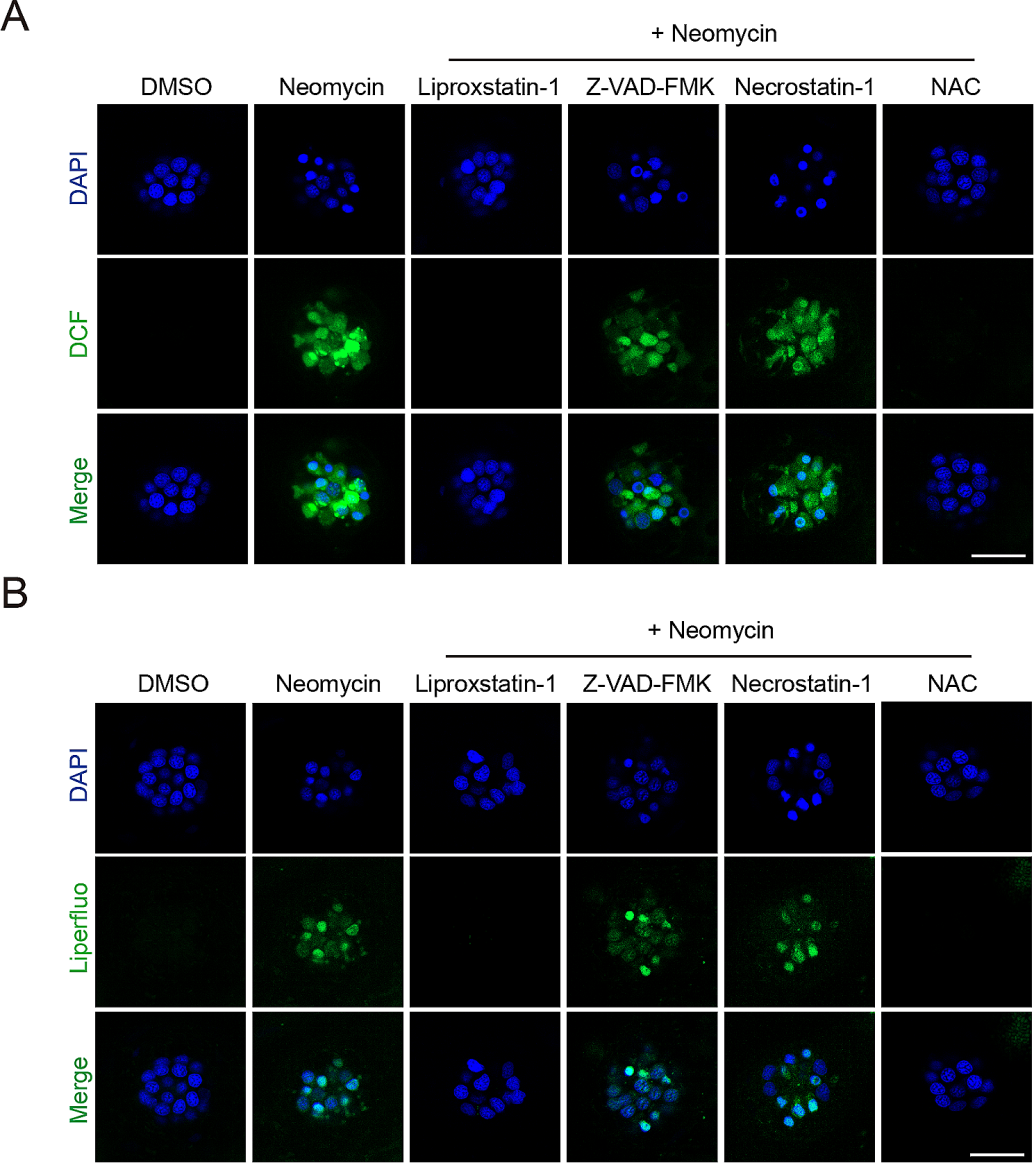
ROS staining in hair cells. Representative confocal images of DCF staining (**A**, for total ROS) or Liperfluo staining (**B**, for lipid ROS) are shown. Neomycin induces the accumulation of total ROS as well as lipid ROS, which can be blocked by Liprosxstatin-1 and NAC, but not Z-VAD-FMK and Necrostatin-1. Assays were repeated twice. Scale bar in **A**-**B**: 20 μm

Accumulation of lipid ROS is a hallmark of ferroptosis. While it can be readily detected in various cell cultures during ferroptosis, in vivo detection of the accumulation of lipid ROS remains challenging. We took advantage of the imaging capacity of zebrafish larvae and investigated whether neomycin induces ROS (total ROS and lipid ROS) accumulation in hair cells in vivo. For detection of total ROS, larvae were first incubated with the dichlorodihydrofluorescein diacetate (DCFH-DA) probe (DCF, 10 min), stimulated with neomycin (125 µM, 15 min) then imaged under a confocal microscope. We found that neomycin treatment clearly induces the accumulation of total intracellular ROS in hair cells which can be scavenged by NAC or Liproxstatin-1 but not Z-VAD-FMK or Necrostatin-1 (Fig. 2A). We then detected lipid ROS by Liperfluo staining and found that neomycin induces a transient accumulation of lipid ROS which is blocked by Liproxstatin-1 or NAC (Fig. 2B). These data further support that neomycin induces ferroptosis in hair cells.

### Neomycin and Fe^3+^ synergistically sensitize cells to ferroptosis through mitochondrial ROS pathway

**Fig. 3:**
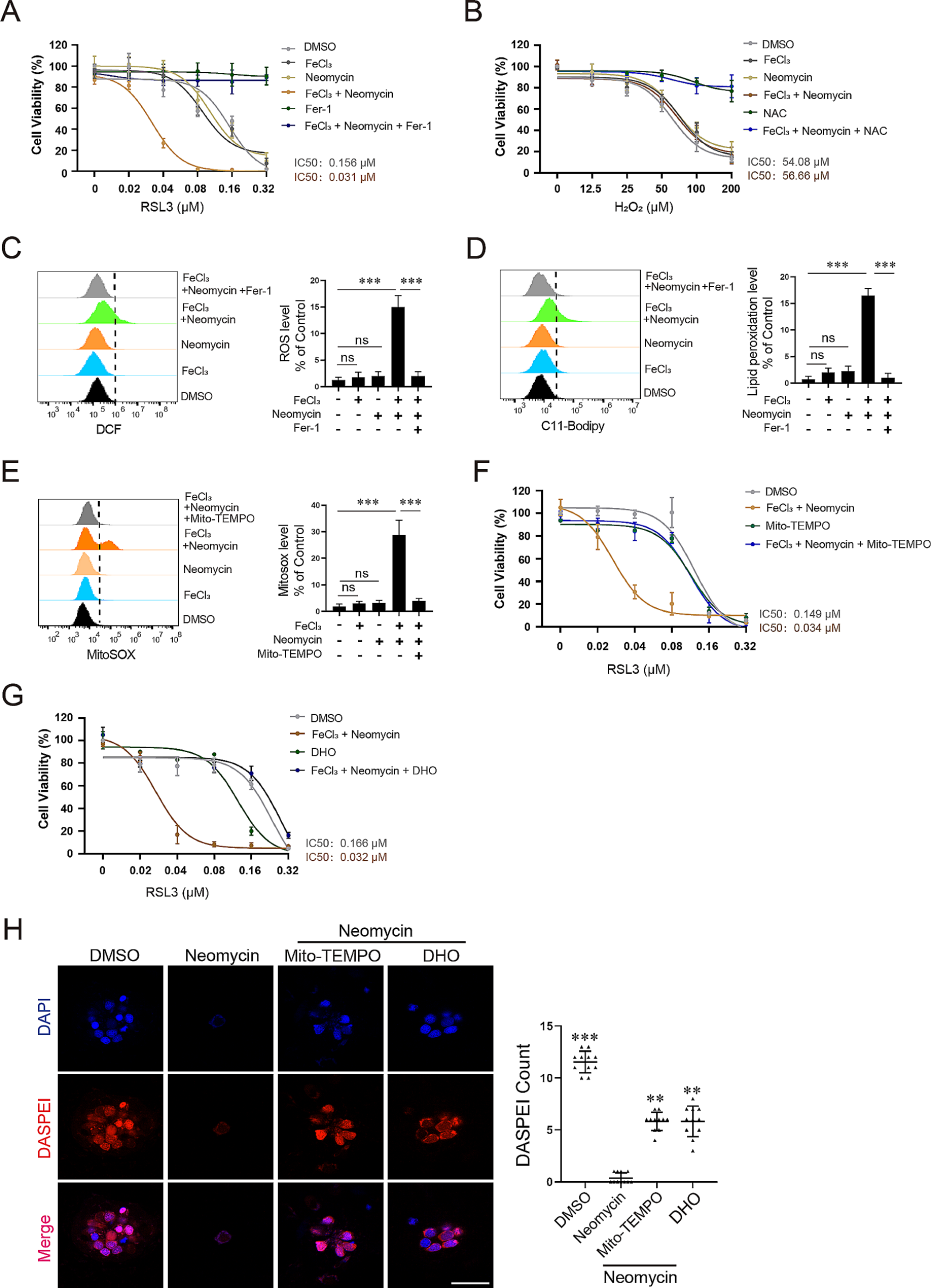
Neomycin and Fe^3+^synergistically sensitize HT1080 cells to ferroptosis inducer RSL3. (**A**) Cell viability assay. Neomycin (500 µM) or Fe^3+^ iron (5 µM) treatment alone has minor effect on ferroptosis sensitivity to RSL3, however, a combination of them dramatically sensitizes HT1080 cells to RSL3-induced ferroptosis. Data represent mean ± s.d. from three independent experiments. (**B**) Neomycin, iron or their combination does not sensitize HT1080 cells to H_2_O_2_-induced cell death. Assays were performed and data were analyzed as described in (**A**). (**C**, **D**) FACS analysis of total ROS (**C**, DCF staining) or lipid ROS (**D**, C11-Bodipy staining) in the treated cells. Neomycin and iron synergistically induce total ROS as well as lipid ROS (in the absence of RSL3), which can be blocked by the ferroptosis inhibitor Fer-1 which is an RTA. Data represent mean ± s.d. from three biological repeats and *p*-value is determined by ordinary one-way ANOVA with Tukey’s multiple comparisons test. ns, no significance; ***, *p* < 0.001. (**E**) Neomycin and iron synergistically induce mitochondrial ROS (MitoSOX staining) which is blocked by the mitochondria-targeted antioxidant Mito-TEMPO. Data represent mean ± s.d. from three biological repeats and *p*-value is determined by ordinary one-way ANOVA with Tukey’s multiple comparisons test. ns, no significance; ***, *p* < 0.001. (**F**) Mito-TEMPO (20 µM) reverses ferroptosis sensitivity to RSL3 in HT1080 cells stimulated by the combination of neomycin and iron. Data represent mean ± s.d. from three independent experiments. (**G**) The neomycin/iron stimulated ferroptosis sensitivity to RSL3 is abolished by DHO treatment (200 µM), which activates the mitochondrial DHODH/CoQ pathway to defense against ferroptosis. Data represent mean ± s.d. from three biological repeats. (**H**) Mito-TEMPO (20 µM) or DHO (200 µM) partially alleviates neomycin-induced hair cell death. Data represent mean ± s.d. and *p*-value is determined by ordinary one-way ANOVA with Dunnett’s multiple comparisons test. **, *p* < 0.01; ***, *p* < 0.001. Scale bar: 20 μm

To investigate the mechanism of neomycin-stimulated ferroptosis, we used the HT1080 cell model which is routinely used in ferroptosis-related studies. We found that neomycin alone (up to 5 mM) does not induce HT1080 cell death (Fig. S1A) and neomycin (200 to 800 µM) does not stimulate ferroptosis sensitivity to RSL3 (Fig. S1B), indicating neomycin alone is not sufficient to stimulate ferroptosis in this cell line. We noticed that a previous biochemical study reported that gentamicin promotes ferric or ferrous ion mediated lipid peroxidation in vitro [42], so we hypothesized that neomycin might synergize with iron to modulate ferroptosis sensitivity in HT1080 cells. We found ferric iron alone (up to 80 µM) does not induce HT1080 cell death (Fig. S1C) and ferric iron at 5–20 µM range has minor effect on ferroptosis sensitivity to RSL3 (Fig. S1D). However, a combination of iron (5 µM) and neomycin (500 µM) dramatically sensitizes HT1080 cells to RSL3-induced cell death (IC_50_ decreased from 156 to 31 nM) (Fig. 3A). Furthermore, this cell death can be fully rescued by ferroptosis inhibitor ferrostatin-1 (Fer-1) (Fig. 3A), indicating it is ferroptosis. Erastin and sorafinib are two other commonly used ferroptosis inducers and we found that the combination of neomycin with iron also sensitizes HT1080 cells to cell death induced by these agents (Fig. S2). We then determined whether neomycin/iron affects cell death induced by hydrogen peroxide (ROS-dependent necroptotic cell death) and found that it does not promote hydrogen peroxide induced necroptosis (Fig. 3B), indicating that iron and neomycin favor the generation of lipid ROS. We next measured the levels of total cellular ROS as well as lipid ROS in control, iron, neomycin, and iron plus neomycin treated cells (all in the absence of RSL3) and found that the combination of iron and neomycin synergistically induces the generation of total and lipid ROS (Fig. 3C, D), which is likely to be responsible for the elevated ferroptosis sensitivity in those treated cells.

AGs are well-established to induce mitochondrial dysfunction and stimulate mitochondrial ROS generation in hair cells which is responsible for AG-induced ototoxicity. So, we determined whether neomycin and iron stimulate ferroptosis through mitochondrial ROS pathways. We found that neomycin (500 µM) or iron (5 µM) alone is not sufficient to stimulate mitochondrial ROS in HT1080 cells, however, a combination of neomycin with iron dramatically stimulates the accumulation of mitochondrial ROS, which can be removed by the administration of mitochondria-targeted ROS scavenger Mito-TEMPO (Fig. 3E). Furthermore, Mito-TEMPO treatment also abolishes neomycin/iron combination induced ferroptosis sensitivity (Fig. 3F). We noticed that Mito-TEMPO does not modulate RSL3 sensitivity in the absence of neomycin/iron (Fig. 3F), indicating that it does not affect RSL3 signaling. To further investigate the involvement of mitochondrial ROS pathway in neomycin/iron stimulated ferroptosis sensitivity, we tested whether dihydroorotate (DHO, 200µM) which protects ferroptosis through the mitochondrial DHODH/CoQ pathway [43] can block neomycin/iron stimulated ferroptosis sensitivity and found that DHO treatment effectively abolishes neomycin/iron stimulated ferroptosis sensitivity (Fig. 3G). We then investigated whether a similar mechanism underlies neomycin-induced hair cell death in vivo. In line with the aforementioned results, we found that both Mito-TEMPO (20 µM) and DHO (200 µM) are able to ameliorate neomycin-induced hair cell death in larvae (Fig. 3H). Together, these data indicate that ferroptosis induced by mitochondrial ROS pathway is responsible for neomycin ototoxicity.

### Chemical screen in zebrafish identifies multiple otoprotective compounds that ameliorate neomycin-induced hair cell death by blocking ferroptosis pathway

**Fig. 4:**
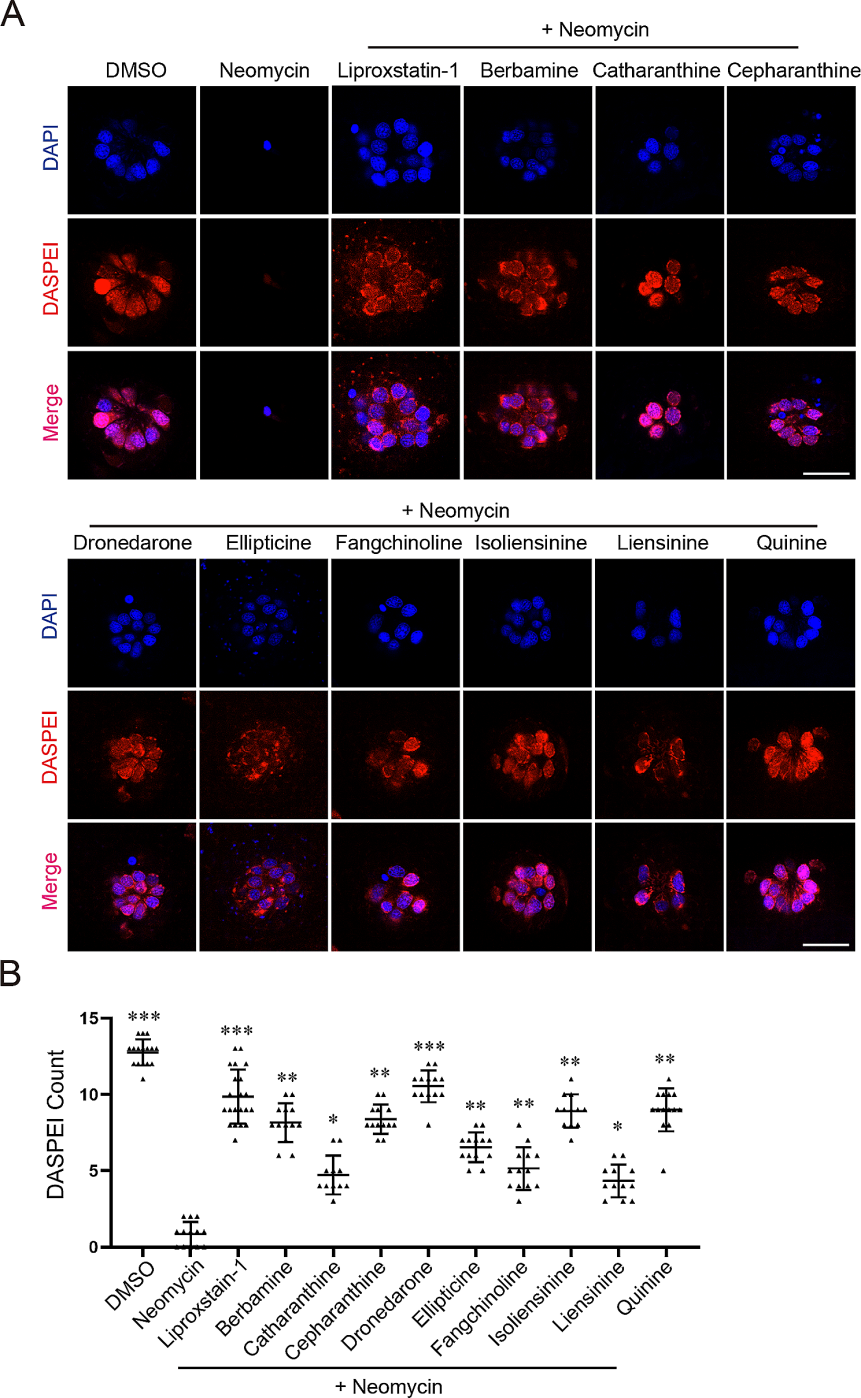
Otoprotection screening against neomycin-induced hair cell damage in zebrafish lateral line. A Representative otoprotection results of the nine positive hit compounds identified from our screen. Liperoxstatin-1 is a positive control. Scale bar: 20 μm. (**B**) Quantitative results for (**A**). Three biological repeats were performed and at least 10 larvae were analyzed for each treatment. *p*-value is determined by ordinary one-way ANOVA with Dunnett’s multiple comparisons test. *, *p* < 0.05; **, *p* < 0.01; ***, *p* < 0.001

**Fig. 5:**
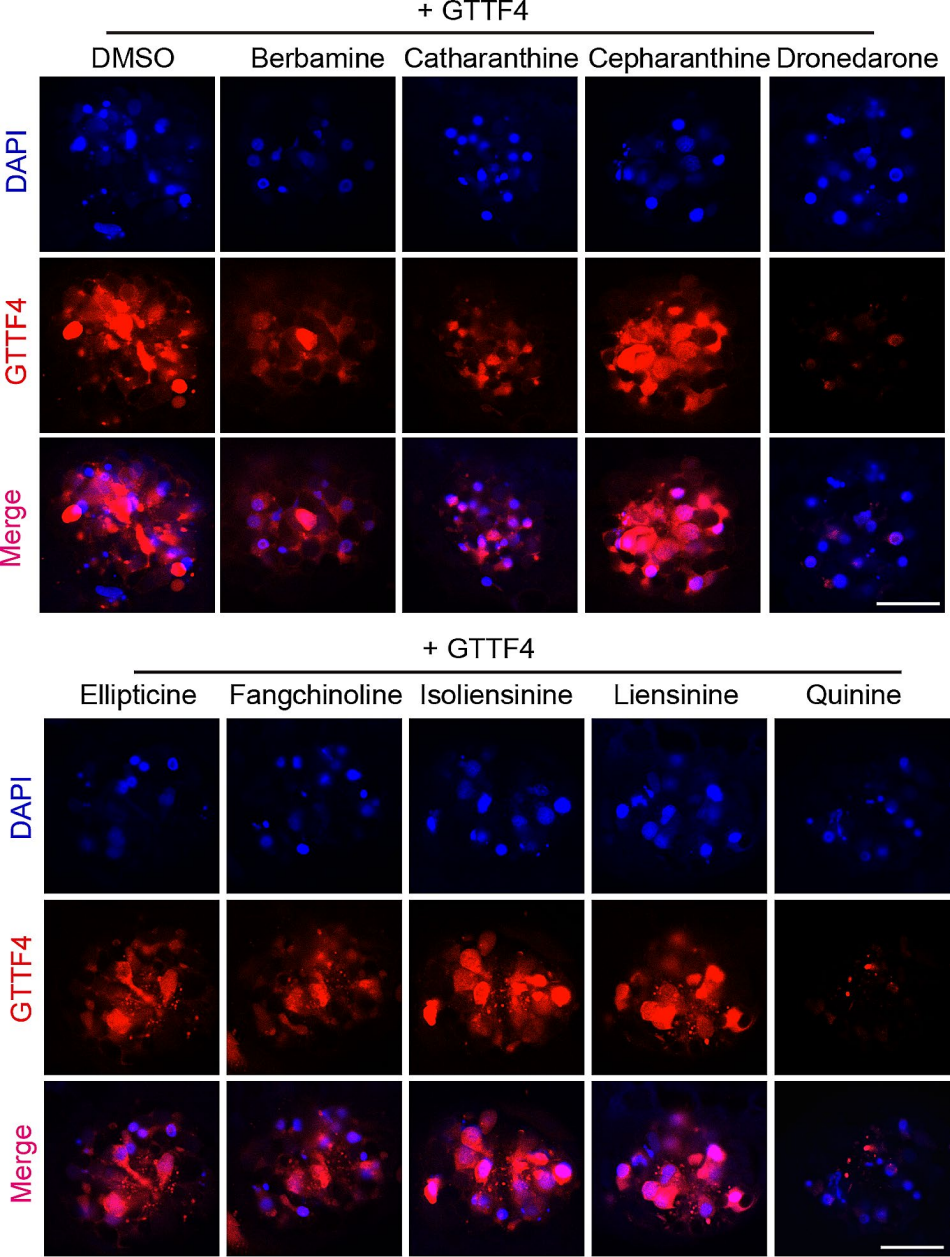
Dronedarone and quinine block the uptake of GTTF4. GTTF4 loading assay were performed to evaluate the ability of a testing compound to block the uptake of GTTF4 in hair cells. Assays were repeated twice, and representative images are shown here. Scale bar: 20 μm

Previous studies have identified many otoprotective compounds against AG-induced hair cell damage and most of them function to block the uptake of AGs [28, 33–35]. Our discovery that neomycin-induced hair cell death is dominated by ferroptosis (unlike gentamicin or cisplatin) indicates that an otoprotection screening under our assay condition might identify additional otoprotective compounds that function to block the ferroptosis pathway. To this end, we screened an alkaloid natural compound library (Table S1) for compounds that can ameliorate neomycin-induced hair cell damage in zebrafish larvae. Briefly, zebrafish larvae at 5 *dpf* were first treated with a testing compound (10 µM) then incubated with neomycin (125 µM) for 2 h. Treated embryos were stained with DASPEI then visually examined under a fluorescence microscope. A typical visual screening result is presented in Fig. S3. Positive hits were retested, and the treated larvae were imaged under a confocal microscope. We totally identified nine compounds with variable otoprotective activities from the screen (Fig. 4A, B). To dissect their otoprotective mechanisms, we first tested whether these compounds block the uptake of AG by GTTF4 loading assay. We found that dronedarone and quinine strongly inhibit the uptake of GTTF4 while other hit compounds show either weak (berbamine and catharanthine) or negligible (cepharanthine, ellipticine, fangchinoline, liensinine and isoliensinine) channel blocker activity (Fig. 5), indicating these compounds have additional otoprotective mechanism(s).

**Fig. 6:**
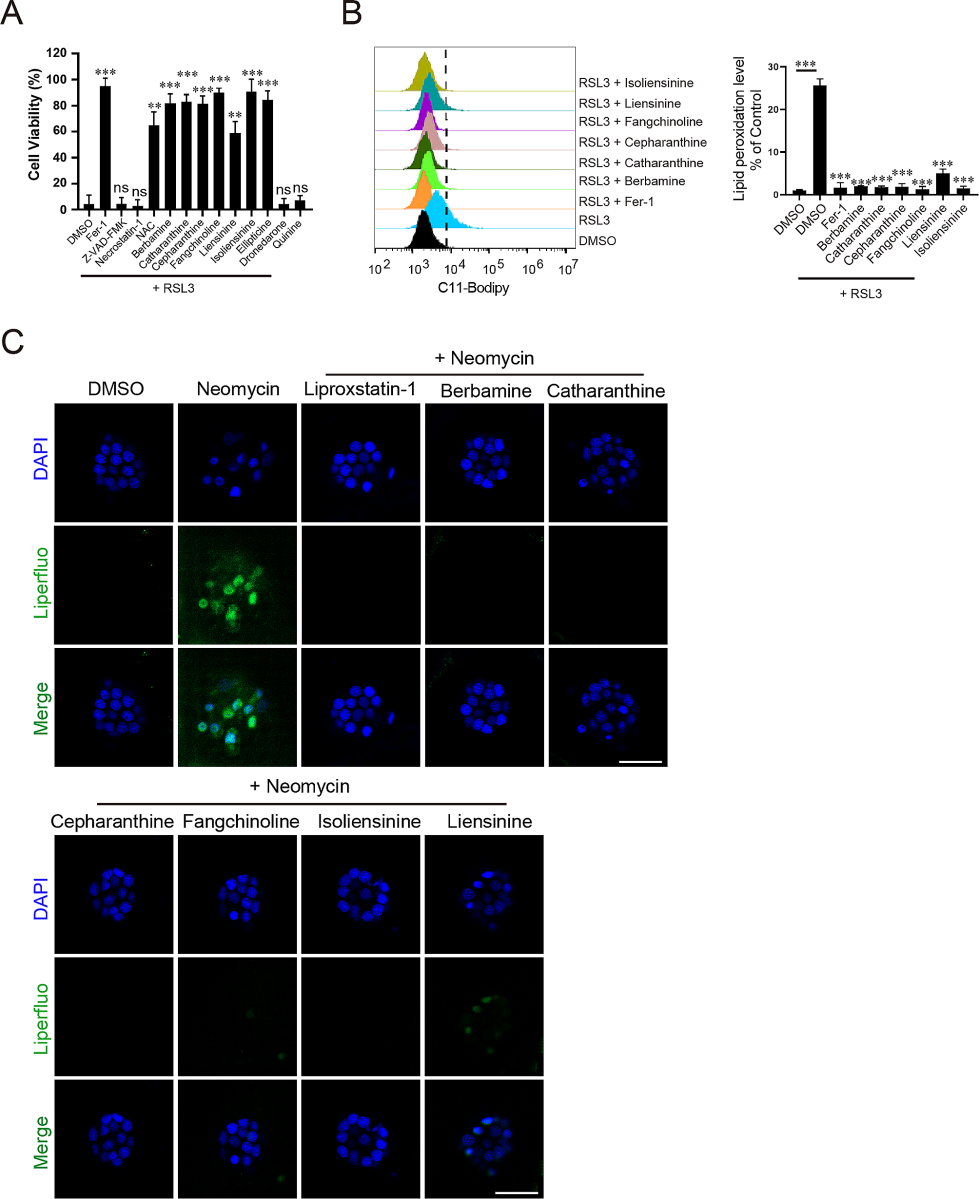
Multiple hit compounds inhibit lipid ROS accumulation and ferroptosis. A All hit compounds other than dronedarone and quinine are able to inhibit RSL3-induced ferroptosis in HT1080 cells. Data represent mean ± s.d. from three biological repeats and *p*-value is determined by ordinary one-way ANOVA with Dunnett’s multiple comparisons test. ns, no significance; **, *p* < 0.01; ***, *p* < 0.001. (**B**) FACS analysis of RSL3-induced lipid ROS in cells pretreated with a hit compound. Data represent mean ± s.d. from three biological repeats and *p*-value is determined by ordinary one-way ANOVA with Dunnett’s multiple comparisons test. ***, *p* < 0.001. (**C**) Hit compounds inhibit neomycin-induced lipid ROS accumulation in hair cells. Assays were performed and analyzed as described in Fig. 2B. Scale bar: 20 μm

We noticed that five of them (berbamine, cepharanthine, fangchinoline, liensinine and isoliensinine) belong to the bisbenzylisoquinoline (BBIQ) compounds that we recently identified as RTAs and ferroptosis inhibitors [44]. Consistent with our previous studies, we found these BBIQ compounds effectively block RSL3-induced ferroptosis in HT1080 cells (Fig. 6A). We then tested whether other hit compounds also have ferroptosis inhibiting activities in HT1080 cells and found that catharanthine and ellipticine can inhibit RSL3-induced ferroptosis while dronedarone and quinine do not have ferroptosis inhibiting activity (Fig. 6A). We next measured their ability to block RSL3-induced lipid ROS accumulation in HT1080 cells (ellipticine was excluded from this assay due to strong autofluorescence) and found that catharanthine as well as the five BBIQ compounds all reduce lipid ROS accumulation (Fig. 6B). We then assayed whether these six compounds have similar ROS scavenging activities in vivo and found that all of them can block neomycin-induced lipid ROS accumulation in hair cells (Fig. 6C). Together, our screen identified nine otoprotective compounds that either block neomycin uptake (2 out of 9) or neomycin-induced ferroptosis (7 out of 9).

### Ellipticine inhibits ferroptosis through down-regulation of TFRC and cell iron content

**Fig. 7:**
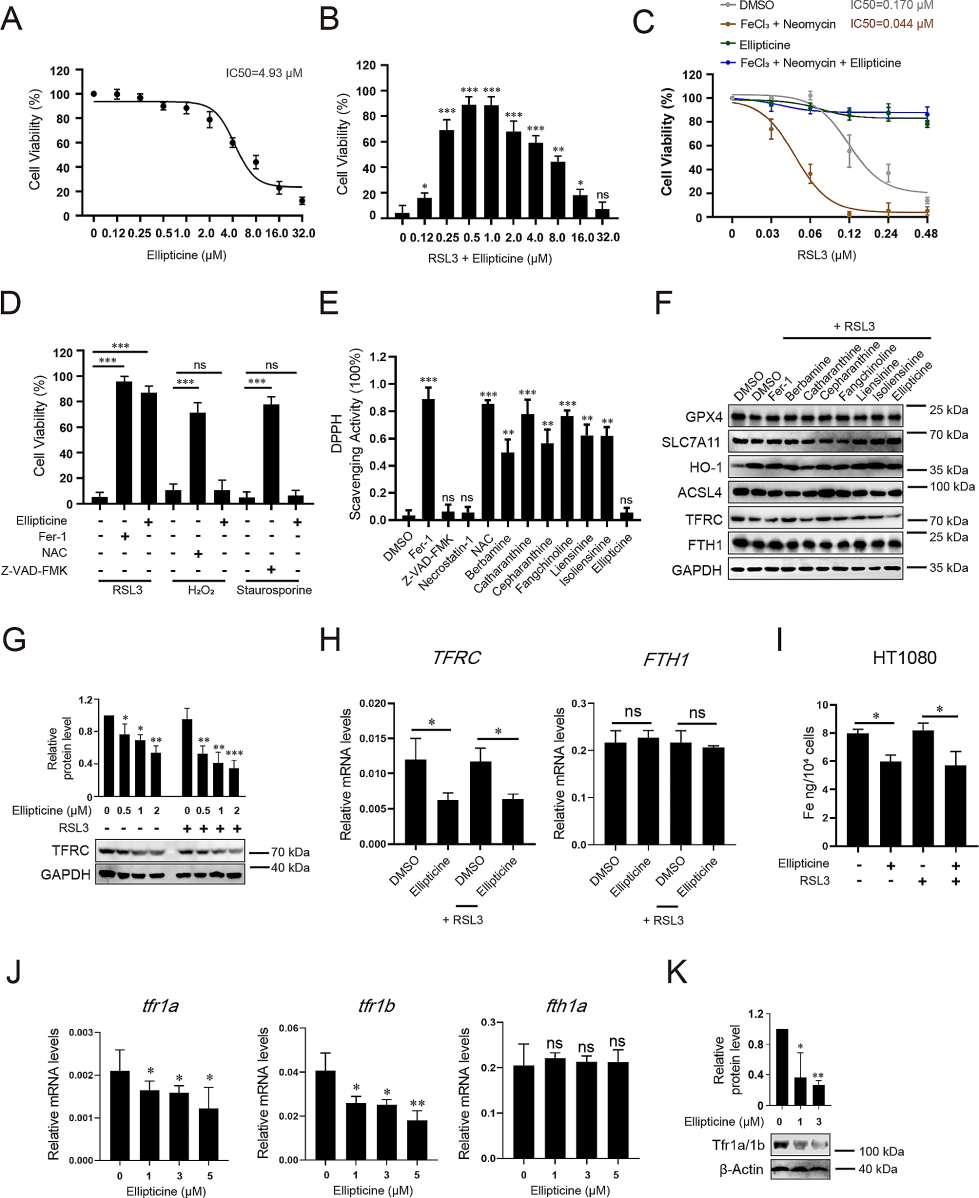
Ellipticine inhibits ferroptosis by downregulation of TFRC and iron homeostasis. A Ellipticine toxicity assay in HT1080 cells. Data represent mean ± s.d. from three biological repeats. (**B**) Protection of RSL3-induced cell death by the indicated concentrations of ellipticine. The best protection is achieved at 0.5 µM. Data represent mean ± s.d. from three repeats and *p*-value is determined by ordinary one-way ANOVA with Dunnett’s multiple comparisons test. ns, no significance; *, *p* < 0.05; **, *p* < 0.01; ***, *p* < 0.001. (**C**) Ellipticine (0.5 µM) protects neomycin/ Fe^3+^ stimulated ferroptosis sensitivity to RSL3. Assays were performed as described in Fig. 3A and data represent mean ± s.d. from three biological repeats. (**D**) Ellipticine protects RSL3-induced (2 µM) ferroptosis, but not hydrogen peroxide-induced (100 µM) necroptosis or staurosporine-induced (2 µM) apoptosis in HT1080 cells. Data represent mean ± s.d. from three independent repeats and *p*-value is determined by ordinary one-way ANOVA with Tukey’s multiple comparisons test. ns, no significance; ***, *p* < 0.001. (**E**) In vitro DPPH assay for ROS scavenging activity. Data represent mean ± s.d. from three repeats and *p*-value is determined by ordinary one-way ANOVA with Dunnett’s multiple comparisons test. ns, no significance; **, *p* < 0.01; ***, *p* < 0.001. (**F**) Western blot for the indicated ferroptosis related proteins. The concentration used is 0.5 µM (for ellipticine) or 5 µM (for all other ferroptosis inhibiting compounds). GAPDH is the loading control. Assays were repeated twice, and representative blots are presented. (**G**) Ellipticine dose-dependently downregulates TFRC in either absence or presence of RSL3 in HT1080 cells. Representative western blots and quantitative results are presented. Data represent mean ± s.d. of 4 blots and *p*-value is determined by ordinary one-way ANOVA with Dunnett’s multiple comparisons test. *, *p* < 0.05; **, *p* < 0.01; ***, *p* < 0.001. (**H**) qRT-PCR analysis for *TFRC* and *FTH1* gene expression in HT1080 cells. *β-actin* is the internal control. Data represent mean ± s.d. of three independent repeats and *p*-value is determined by student’s t-test. ns, no significance; *, *p* < 0.05. (**I**) Cell iron content assay in HT1080 cells. Data represent mean ± s.d. of three independent assays and *p*-value is determined by student’s t-test. *, *p* < 0.05. (**J**) qRT-PCR analysis for zebrafish *tfr1a*, *tfr1b* and *fth1a* gene expression. *β-actin* is the internal control. Data represent mean ± s.d. of three independent repeats and *p*-value is determined by ordinary one-way ANOVA with Dunnett’s multiple comparisons test. ns, no significance; *, *p* < 0.05; **, *p* < 0.01. (**K**) Western blot for transferrin receptor expression in zebrafish total embryonic lysate. β-Actin is the loading control. A representative blot and quantitative results are presented. Data represent mean ± s.d. of 3 blots and *p*-value is determined by ordinary one-way ANOVA with Dunnett’s multiple comparisons test. *, *p* < 0.05; **, *p* < 0.01

Ellipticine is a natural compound that inhibits DNA topoisomerase II and stimulates cell death in a variety of tumor cell lines, which appears contradictory to the anti-cell death activity we report here. So, we further investigated ellipticine in cell viability assay. We found that ellipticine dose-dependently induces HT1080 cell death at micromolar level (IC_50_ = 4.93 µM) (Fig. 7A) while it is able to inhibit RSL3-induced ferroptosis at sub-micromolar level (peaked at 0.5 µM, Fig. 7B). It also blocks neomycin/Fe^3+^ stimulated ferroptosis sensitivity to RSL3 (Fig. 7C). The anti-cell death activity of ellipticine is ferroptosis-specific as it does not block hydrogen peroxide induced necroptosis or staurosporine-induced apoptosis in the same cell line (Fig. 7D). We evaluated ROS scavenging capacity of ellipticine by in vitro DPPH assay and found it has no ROS scavenging activity (Fig. 7E), indicating it inhibits ferroptosis by mechanism(s) other than RTA.

To investigate the anti-ferroptotic mechanism of ellipticine, we first analyzed whether ellipticine affects protein levels of key ferroptosis regulators by western blot. We found that the protein levels of GPX4, SLC7A11, HO-1, ACSL4 and FTH1 are not affected by ellipticine treatment (Fig. 7F), however, transferrin receptor (TFRC, a cell surface receptor essential for cellular iron uptake) is dose dependently down-regulated upon ellipticine treatment either in the absence or presence of RSL3 (Fig. 7F, G). We then measured the mRNA level of *TFRC* by qRT-PCR and found that ellipticine treatment reduces *TFRC* mRNA in either unstimulated or RSL3-stimulated cells (Fig. 7H). On the contrary, the mRNA level or protein level of *FTH1* (heavy chain of ferritin, which is the major intracellular iron storage protein) is unaffected by ellipticine treatment (Fig. 7F, H), indicating ellipticine does not affect iron metabolism globally. We then measured intracellular iron content and as expected, down-regulation of TFRC reduces cell iron content in ellipticine-treated cells (Fig. 7I). We also investigated whether a similar mechanism operates in zebrafish. Zebrafish has two transferrin receptor gene homologs (*tfr1a* and *tfr1b*) and we found ellipticine treatment reduces mRNA levels of both (Fig. 7J). Consistent with this, the protein level of transferrin receptors in total embryonic lysate is also reduced after ellipticine treatment (Fig. 7K). Together, these results indicate that ellipticine protects ferroptosis by downregulating *TFRC* expression and reducing cellular iron content in both HT1080 cells and zebrafish embryos.

## Discussion

Zebrafish lateral line provides an accessible model to study hair cell development/ regeneration, or screen compounds for either ototoxic or otoprotective activities. Hair cell damages can be induced by a variety of ototoxic reagents including copper, cisplatin, or AGs while their underlying mechanisms are not fully understood. CuSO_4_ has been reported to induce oxidative stress and necrotic hair cell death and it can be blocked by antioxidants [45], which is consistent with our finding here that Necrostatin-1 or NAC blocks CuSO_4_ induced hair cell damages (Fig. 1F). Surprisingly, we found here that ferroptosis appears dispensable for CuSO_4_ induced hair cell death, indicating that oxidative stress in hair cells does not necessary lead to ferroptosis.

Cisplatin is a widely used chemotherapeutic reagent and ototoxicity is a major side effect in patients. Cisplatin-induced hair cell damages have been well documented in zebrafish. In a recent study, it is reported that the ferroptosis inhibitor Fer-1 protects cisplatin-induced ototoxicity in zebrafish hair cells [46] which appears contradictory to our result that the ferroptosis inhibitor Liproxstatin-1 fails to protect cisplatin-induced hair cell death (Fig. 1E). Although both Fer-1 and Liproxstatin-1 show excellent ferroptosis inhibiting activity in cell cultures, Fer-1 is generally reported to be less effective and more toxic in vivo as ferroptosis inhibitor. In our hand, Fer-1 at 10, 20, or 40 µM all fails to block neomycin-induced hair cell death (data not shown) while Liproxstatin-1 at 10 µM is sufficient to protect neomycin ototoxicity (Fig. 1A). Thus, the inability of Liproxstatin-1 to block cisplatin-induced hair cell death cannot be attributed to Liproxstatin-1 as a poor ferroptosis inhibitor in vivo, instead, it is more likely that ferroptosis does not contribute significantly to cisplatin-induced hair cell death under our assay condition. We notice that different cisplatin treatment protocols are used in these two studies (200 µM for 2 h vs. 40 µM for 24 h) which may lead to the activation of different intracellular death pathways and the observed inconsistency.

We revealed that ferroptosis is involved in hair cell damages induced by all AGs tested (neomycin, streptomycin, gentamycin, and amikacin) while apoptosis and necroptosis is activated by gentamicin and amikacin but not neomycin or streptomycin in these assays, indicating the activation of distinct intracellular death pathways by these AGs. Indeed, differences between neomycin- and gentamicin-induced hair cell deaths in zebrafish larvae have been reported in several previous studies. For example, neomycin-induced acute hair cell death is fully protected by pretreatment with the compound PROTO1 while gentamicin appears to induce hair cell death in biphasic manner: an acute phase that can be protected by PROTO1 and a chronic phase that cannot be protected by PROTO1 [47]. On the other hand, d-methionine treatment or overexpression of Bcl2 is able to block gentamicin but not neomycin induced ototoxicity [48, 49]. It will be interesting to test whether ferroptosis is responsible for these observed differences.

Inhibition the uptake of AGs is an effective way to protect AG-induced ototoxicity in zebrafish as well as other models. Small Compound screening in zebrafish have identified many otoprotectants that function to block the uptake of AGs. Our discovery that ferroptosis is the dominant cell death in neomycin-induced hair cell damage in zebrafish indicates that inhibition of ferroptosis could be another effective way to ameliorate neomycin-induced ototoxicity. To test this hypothesis, we performed a compound screening of alkaloid natural compound library which is known to be rich in antioxidants. We identified nine otoprotective compounds from this screen and two of them function to block neomycin uptake and seven of them inhibit ferroptosis, supporting an otoprotective function upon ferroptosis inhibition. Among the seven ferroptosis inhibiting compounds, berbamine, cepharanthine, fangchinoline, liensinine and isoliensinine are BBIQ compounds that were recently identified as ferroptosis inhibitors in our cell-based screen [44]. Catharanthine does not contain the chemical backbone of BBIQ compounds, but it also has ROS scavenging activity (Fig. 7D) and shows ferroptosis inhibiting activity in cell-based assay (Fig. 6A). These data indicate that a compound’s ferroptosis inhibiting activity in zebrafish hair cells largely predicts its activity in cell-based assay. Meanwhile, 15 out of the top 20 hits from our cell-based screen [44] are not otoprotective in vivo in neomycin-induced hair cell death assay, indicating ferroptosis inhibiting activity in cell-based assay does not guarantee otoprotective function in vivo, possibly due to factors such as compound stability, absorption, distribution, metabolism, toxicity, etc. Thus, positive hits from cell-based screen should be further validated in vivo in models such as zebrafish or mouse for their potential as candidate compounds for further drug development.

We noticed one compound, ellipticine, is not a top positive hit in our previous cell-based screen but protects neomycin-induced hair cell death in vivo. We further analyzed this compound and found that at the dose used in the previous screen (5 µM) it is toxic to HT1080 cells, which prevents it be identified as a positive hit in the previous screen (Fig. 7B). We now show that ellipticine at sub-micromolar level is sufficient to inhibit ferroptosis in HT1080 cells via a unique mechanism: instead of scavenging lipid ROS, it downregulates intracellular iron content by inhibiting TFRC expression thus reduces the iron-dependent generation of lipid ROS. Downregulation of transferrin receptor by ellipticine is also observed in zebrafish embryos. Thus, it will be interesting to test whether ellipticine, by downregulating TFRC and cell iron content, has beneficial effects in the treatment of other iron-overload related disorders such as iron storage diseases.

In conclusion, our study reveals that neomycin stimulates ferroptosis in zebrafish lateral line hair cells as well as in in vitro cultured cell lines through mitochondrial ROS pathway. Ferroptosis inhibition by stimulating the mitochondrial anti-ferroptotic DHODH/CoQ pathway, reducing intracellular iron content and lipid ROS generation, or promoting lipid ROS scavenging all show otoprotective activities. These results support the idea that, in addition to blocking the uptake of AGs, targeting intracellular death pathway can also be utilized to ameliorate AG-induced ototoxicity.

## Materials and methods

### Zebrafish ototoxicity assay

Zebrafish were maintained according to standard protocols (zfin.org) and AB strain wildtype zebrafish were used for all experiments. Animal experimental protocols were approved by the Institutional Animal Care and Use Committee at Guangzhou Institutes of Biomedicine and Health.

For otoprotection assay, zebrafish larvae at 5 *dpf* were cultured in 24-well plate (5–8 larvae per well in 1 ml E3 media (5 mM NaCl, 0.17 mM KCl, 0.33 mM CaCl_2_ and 0.33 mM MgSO_4_, pH 7.2)), then pretreated with Liproxstatin-1 (Selleck, S7699, 10 µM), Z-VAD-FMK (Selleck, S7023, 50 µM), Necrostatin-1 (Selleck, S8037, 50 µM) or NAC (Selleck, S1623, 5 mM) for 2 h, then treated with neomycin (Selleck, S2568, 125 µM), streptomycin (Selleck, S2572, 125 µM), gentamicin (Selleck, S4030, 125 µM), amikacin (Selleck, S3122, 125 µM), cisplatin (Selleck, S1166, 200 µM), or CuSO_4_ (Macklin, C805233, 200 µM) for 2 h at 28℃. An alkaloid natural product library from TargetMol (TargetMol, Shanghai, China, L6110, 10 µM) were used to screen for compounds that can protect neomycin-induced hair cell death in zebrafish larvae. After treatment, larvae were washed with E3 media for 3 times, then stained with DASPEI (AAT Bioquest, 22225, 1 µM) and DAPI (Beyotime, C1005, 0.2 µg/mL) for 15 min at 28℃. Larvae were then washed with E3 media, embedded in 5% low-melting agarose then imaged using the Zeiss LSM800 confocal microscope. Assays were repeated two to three times and at least ten larvae were analyzed for each treatment.

### ROS staining in larvae

For total ROS staining, larvae were treated with the indicated compounds as described above, washed with E3 media, then incubated in E3 containing DCFH-DA (Beyotime, S0033S, 10 µM) and DAPI (0.2 µg/mL) for 10 min in dark. Larvae were washed with E3, stimulated with 125 µM neomycin for 15 min, washed again with E3, then embedded in 5% low-melting agarose and ready for imaging using the Zeiss LSM800 confocal microscope. For lipid ROS staining, larvae were first incubated in Liperfluo (Dojindo, L248, 1 µM) and DAPI (0.2 µg/mL) for 20 min in dark. Larvae were then washed, then stimulated with neomycin (125 µM, 5 min), then imaged as described above.

### GTTF4 loading assay

Fluorescent labeling of gentamicin was performed by mixing Tide Fluor™ 4 (TF4, AAT Bioquest, 2289, 10 mg/mL in DMSO) with gentamicin (Topscience, T1362, 100 mM) (1:5 v/v) for 6–12 h at RT in dark which generates gentamicin-TF4 conjugate (GTTF4, 83 mM final concentration). Larvae were pretreated with the indicated testing compound (10 µM for 2 h), washed with E3, then incubated with GTTF4 (final concentration of 125 µM) and DAPI (0.2 µg/mL) for 15 min at 28℃. After incubation, larvae were washed with E3, fixed in 4% PFA (Beyotime, P0099) for 30 min at 4℃. Samples were then embedded in 5% low-melting agarose and imaged using the Zeiss LSM800 confocal microscope.

### DPPH assay

The free radical scavenging capacity of a testing compound was assayed using the DPPH Assay Kit (Solarbio, BC4755). Briefly, NAC was diluted in the provided solution to the final concentration of 5 mM and all other testing compounds were diluted to 100 µM. An aliquot of testing compound solution (5 µL) was mixed thoroughly with 195 µL of DPPH working solution in 96-well plate and incubated in dark for 30 min at RT. After reaction, the absorbance (A) of the reaction mixture was assayed at the wavelength of 515 nm using an automatic microplate reader (Thermo, Epoch 2). The DPPH scavenging rate is calculated as: DPPH Scavenging Activity = [[A blank - (A sample - A control)]/A blank] × 100%.

### Cell viability assay

HT1080 cells were maintained in DMEM High Glucose cell culture medium (Gibco, 11995-065) supplemented with 10% fetal bovine serum (Gibco, 25,200,072) and 1% Penicillin-Streptomycin Solution (Solarbio, P1400) at 37℃ with 5% CO_2_. For rescuing assay, cells were cultured in 96-well plate (1 × 10^4^ cells per well) for 24 h, then stimulated with RSL3 (Selleck, S8155, 2 µM) together with a testing rescuing compound (Liproxstatin-1, 10 µM; Z-VAD-FMK, 50 µM; Necrostatin-1, 50 µM; NAC, 5 mM; other testing compound, all at 10 µM) for 24 h at 37℃. After the incubation, cell viability was assayed with the Cell Counting Kit-8 (CCK-8) (MCE, HY-K0301) according to the manufacturer’s protocol. Assays were performed in triplicates and repeated three times.

### Flow cytometry analysis of ROS

HT1080 cells were seeded on 12-well plates at the density of 1 × 10^5^ cells per well overnight then treated with the indicated compounds for 24 h. For detection of total ROS, cells were washed with serum-free DMEM then incubated in DCFH-DA probe (Beyotime, Shanghai, China, S0033S, 10 µM in serum-free medium) for 30 min at 37˚C in dark. For detection of lipid ROS, treated cells were washed then incubated in BODIPY™ 581/591 C11 lipid ROS probe (Thermo, D3861, 2 µM in serum-free media) for 1 h in dark at 37℃. For detection of mitochondrial ROS, cells were stained in MitoSOX™ Red (Thermo, M36008, 5 µM in serum-free media) for 1 h in dark at 37℃. After dye incubation, cells were washed 3 times with serum-free medium, trypsinized with 200 µL 0.25% Trypsin-EDTA (Gibico, 25,200,056). Trypsinization was terminated by 5% FBS/PBS and cells were collected by centrifugation at 1300 rpm for 3 min. Cells were resuspended in sterile 5% FBS/PBS, filtrated (70-µm net), then analyzed using the BD Accuri C6 Plus Flow Cytometer (BD). Data were analyzed with the BD FlowJo V10 software.

### Western blot

HT1080 cells were cultured in 24-well plates (1 × 10^5^ cells per well) overnight then treated with the indicated compounds for 24 h as described above. After compound treatment, cells were lysed in 100 µL of lysis buffer (a 4:1:0.01 mixture of Cell Lysis Buffer (Beyotime, P0013), 5×Loading Buffer (Beyotime, P0015L) and PMSF (Beyotime, ST506)) on ice for 5 min. For western blot analysis of zebrafish sample, embryos (1 *dpf*) were treated with ellipticine for 24 h then lysed in 150 µL of lysis buffer on ice for 10 min. The mixture was then transferred to an EP tube and incubated in boiling water for 5 min. Samples were then centrifuged at 5,000 rpm for 1 min and supernatants were separated by 12% SDS-PAGE. After transferred to PVDF membranes (EMD Millipore, MA, IPVH00010), samples were blocked in solution (5% skim milk in TBST) for 1 h, washed with TBST for 3 times, then incubated with a primary antibody diluted in QuickBlock™ Primary Antibody Dilution Buffer for Western Blot (Beyotime, P0256) overnight at 4 ℃. Blots were then washed with TBST and incubated with a HRP-conjugated secondary antibody (Goat Anti-Mouse IgG (H + L), Proteitech, Wuhan, China, SA00001-1, 1: 5000; or Goat Anti-Rabbit IgG (H + L), Proteitech, SA00001-2, 1: 5000) for 2 h at RT. Blots were then washed with TBST extensively, detected with the Immobilon Western Chemiluminescent HRP Substrate (EMD Millipore, WBKLS0100) and imaged using the GelView 6000 plus imaging system (BTL). Primary antibodies used for cell-based assay (HT1080) are: anti-GPX4 (1:1000, Abcam, Cambridge, UK, ab125066), anti-SLC7A11 (1:1000, Proteintech, 26864-1), anti-ACSL4 (1:1000, Abcam, ab205199), anti-FTH1 (1:1000, Abcam, ab75973), anti-TFRC (1:1000, Abcam, ab84036), anti-GAPDH (1:1000, Proteintech, HRP-60,004). Antibodies for zebrafish embryonic lysate are anti-Tfr1a/b (1:2000, Proteintech, 66180-1-lg) and anti-β-Actin (1:1000, Proteintech, 66009-1-lg).

### RNA extraction and qRT-PCR

Cells were cultured on 12-well plates and treated with the indicated compounds for 24 h. Zebrafish embryo (1 *dpf*) were treated with the indicated doses of ellipticine for 24 h. Total RNA was prepared with the FastPure Cell/Tissue Total RNA Isolation Kit V2 (Vazyme, RC112-01) according to manufacturer’s protocol. cDNA was synthesized using the HiScript Q RT SuperMix for qPCR Kit (Vazyme, R122-01) and PCR reaction was performed using the ChamQ SYBR qPCR Master Mix (Vazyme, Q331-02) on the CFX96 Touch Real-Time PCR Detection System (BioRad, CA). qPCR primers for human genes are: TFRC-F: ATCGGTTGGTGCCACTGAATGG; TFRC-R: ACAACAGTGGGCTGG CAGAAAC; β-Actin-F: CACCATTGGCAATGAGCGGTTC; β-Actin-R: AGGTCTTTGCGGATGTCCACGT. qPCR primers for zebrafish genes are: tfr1a-F: GGCAGGACTGGTCACTCTTC; tfr1a-R: CCTGATCTGGGACACGTAGC; tfr1b-F: GGACTCTGTCACTGGTCAGC; tfr1b-R: GTCATGAACGAGACGGAGGG; fth1a-F: GAACAAGAGAGGTGGACGCA; fth1a-R: CAGCCCATTGTCCCACTCAT; β-Actin-F: GGTATCGTGCTGGACTCTGG; β-Actin-R: GTGTGGCAGAGCATAACCCT.

### Cell iron content assay

Cell iron concentration was detected with the Cell Iron Content Assay Kit according to manufacturer’s protocol (Solarbio, BC5315). Briefly, 2.5 × 10^6^ cells were treated with the indicated compounds for 24 h, trypsinized with 500 µL 0.25% Trypsin-EDTA then terminated by 5% FBS DMEM High Glucose cell culture medium. Cells were collected by centrifugation at 1300 rpm for 3 min. Cells were treated with 500 µL extraction reagent and ultrasonificated for 1 min, centrifuged at 10,000 rpm for 10 min. An aliquot of supernatant (20 µL) was mixed with iron detection reagent for 10 min at RT then absorbance measured at 510 nm. Cell iron content (ng/10,000 cell) = 27.922 × (A sample - A control) ÷ (A standard-sample - A control).

### Statistical analyses

Statistical analyses were performed using the GraphPad Prism 8.0.2 software (GraphPad Software, CA). Data were collected from 3 independent biological repeats and expressed as means ± s.d. Statistical significance was determined using student’s t-test, ordinary one-way or two-way ANOVA, and *p*-value < 0.05 were considered statistically significant.

### Electronic supplementary material

Below is the link to the electronic supplementary material.

Supplementary Material 1

Supplementary Material 2

## Data Availability

All data generated or analyzed during this study are included in this published article and its supplementary information files.

## Abbreviations

AGs: Aminoglycoside antibiotics
ROS: Reactive oxygen species
RTAs: Radical trapping agents
*dpf*: *Day post fertilization*
NAC: N-acetylcysteine
DASPEI: 2-[4-(Dimethylamino)styryl]-1-ethylpyridinium iodide
DAPI: 4’,6-Diamidino-2-phenylindole
DCFH-DA: Dichlorodihydrofluorescein diacetate
DHO: Dihydroorotate
BBIQ: Bisbenzylisoquinoline
GTTF4: Gentamicin-TF4 conjugate
TFRC: Transferrin receptor
FTH1: Heavy chain of ferritin
