# Supplementary figures and images for: Chemical screen in zebrafish lateral line identified compounds that ameliorate neomycin-induced ototoxicity by inhibiting ferroptosis pathway
Source: Cell Biosci. 2024 Jun 5;14:71. doi: 10.1186/s13578-024-01258-w (PMC11151469; doi:10.1186/s13578-024-01258-w)

Figure 7F

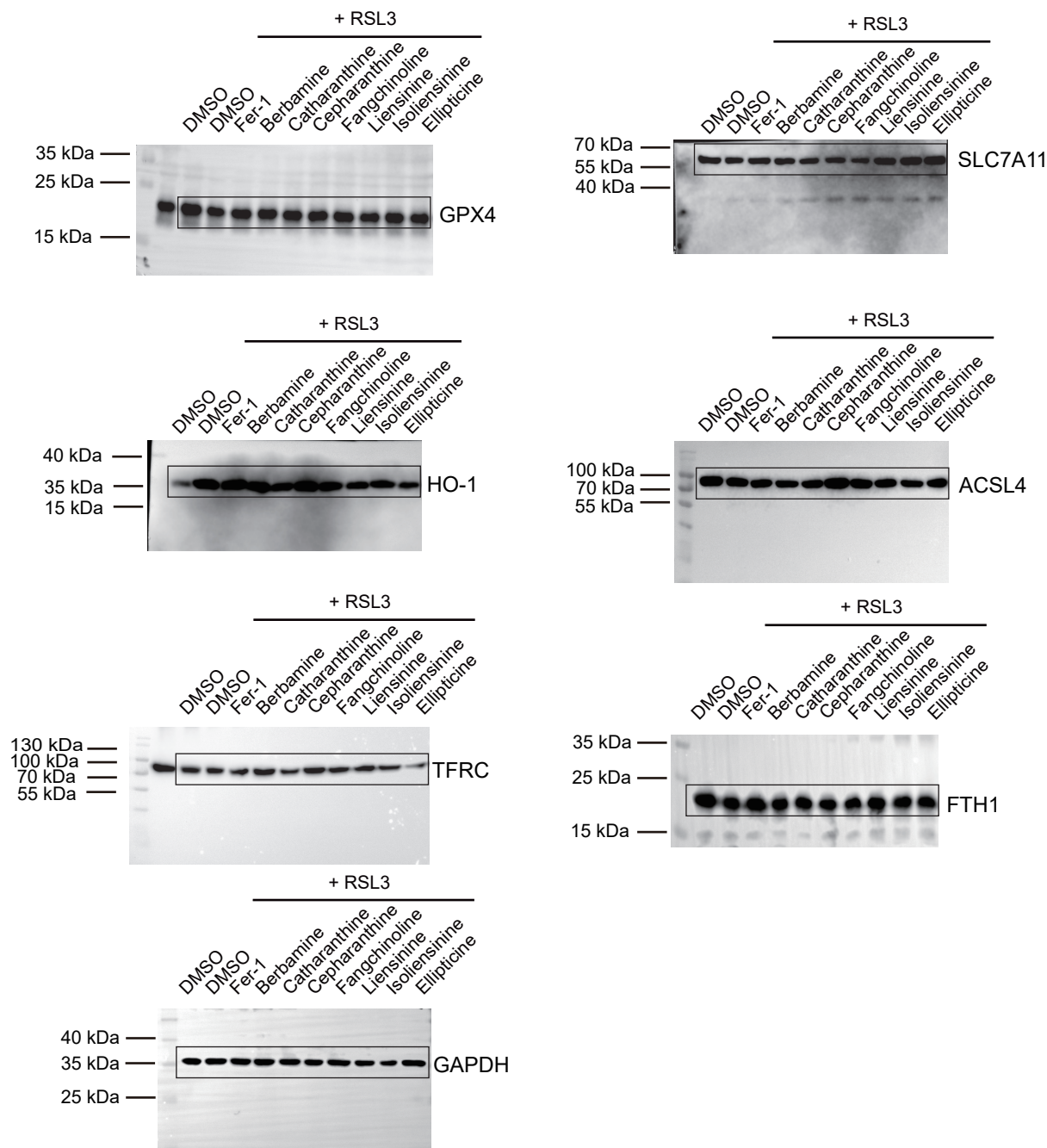

Figure 7G

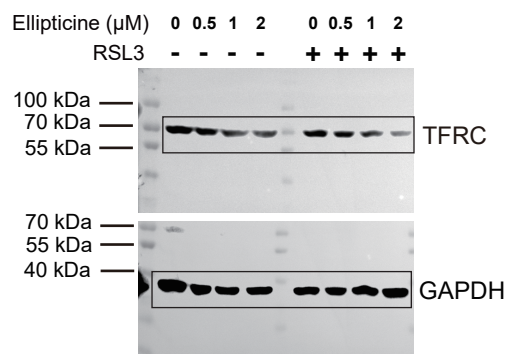

Figure 7K

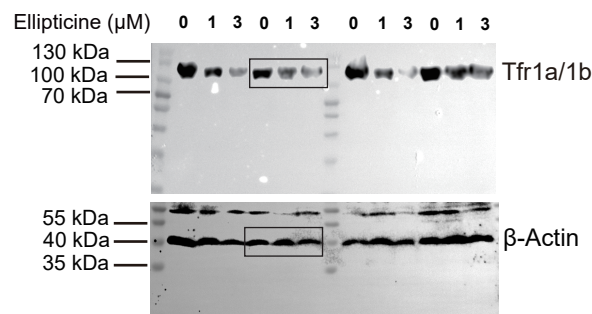

Supplement: Supplementary file 1 — Supplementary Material 1 [file 13578_2024_1258_MOESM1_ESM.pdf]
