# Supplementary material for: Chemical screen in zebrafish lateral line identified compounds that ameliorate neomycin-induced ototoxicity by inhibiting ferroptosis pathway
Source: Cell Biosci. 2024 Jun 5;14:71. doi: 10.1186/s13578-024-01258-w (PMC11151469; doi:10.1186/s13578-024-01258-w)
Supplement: Supplementary file 2 — Supplementary Material 2 [file 13578_2024_1258_MOESM2_ESM.docx]

Supplementary Materials for

**Chemical screen in zebrafish lateral line identified compounds** **that ameliorate neomycin-induced ototoxicity by inhibiting ferroptosis pathway**

Yipu Fan *et al.*

**This supplementary file includes:**

**Fig. S1**. Cell viability assay in HT1080 cells

**Fig. S2**. Neomycin and Fe3+ synergistically sensitizes HT1080 cells to erastin- or sorafenib-induced cell death.

**Fig. S3**. Visual screening for otoprotectants against neomycin-induced hair cell death in zebrafish larvae

**Tables S1**. List of alkaloid natural compounds used in the screen

**
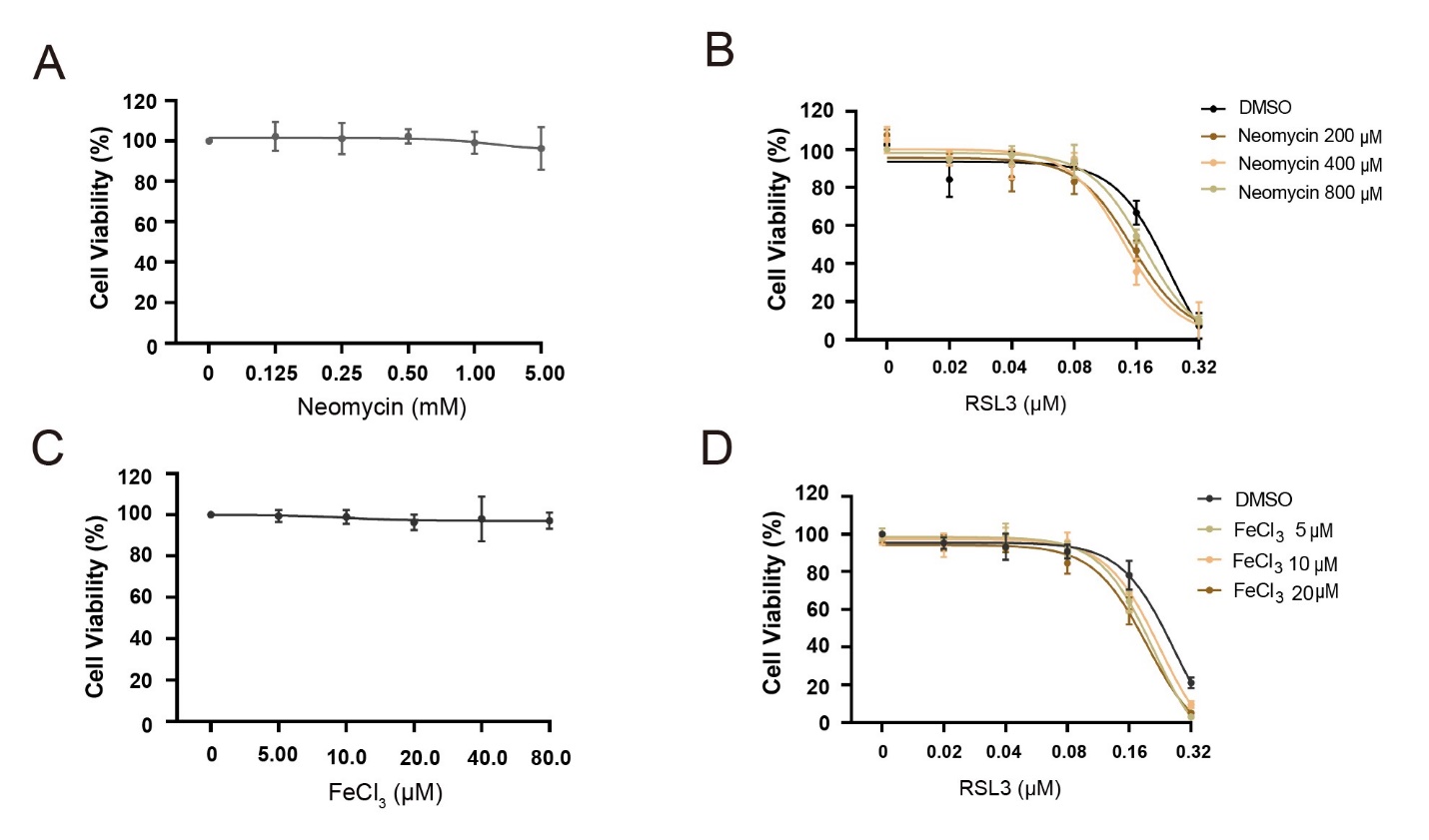
 Fig. S1. Cell viability assay in HT1080 cells.** **A** Neomycin treatment at 0-5 mM range does not affect HT1080 cell viability. **B** Neomycin (200-800 µM) does not sensitize HT1080 cells to RSL3-induced ferroptosis. **C** Fe^3+^ (0-80 µM) does not induce HT1080 cell death. **D** Fe^3+^ (5-20 µM) does not significantly enhance ferroptosis sensitivity to RSL3 in HT1080 cells. Data represent mean ± s.d. of three biological repeats.


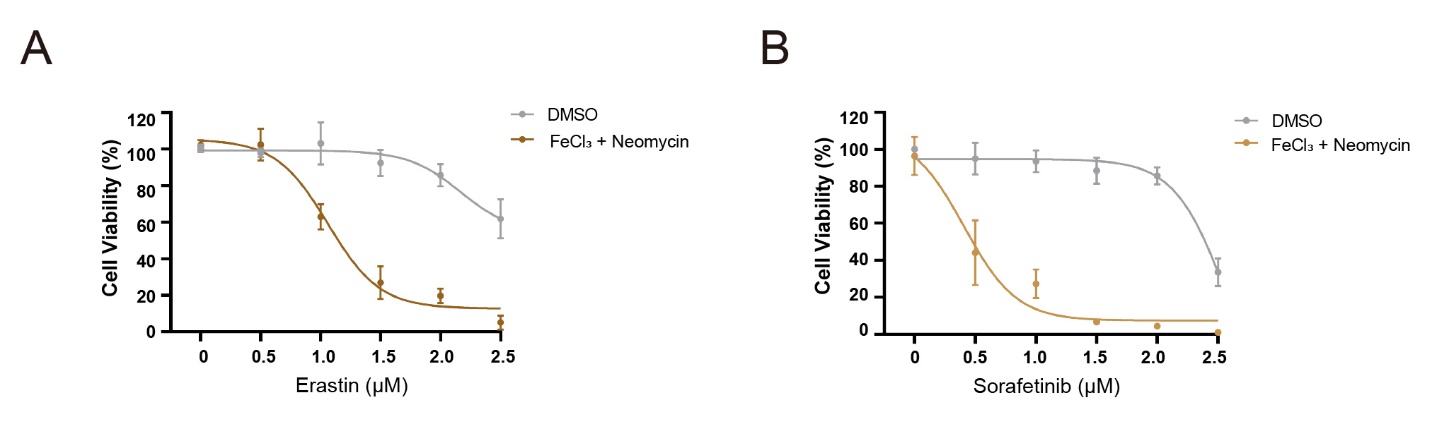


**Fig. S2. Neomycin and Fe^3+^ synergistically sensitizes HT1080 cells to erastin- or sorafenib-induced cell death.** Pretreatment of HT1080 cells with a combination of neomycin (500 µM) and Fe^3+^ (5 µM) stimulates sensitivity to either erastin (**A**) or sorafenib (**B**) induced cell death. Assays were performed as described in **Fig. 3A** and data represent mean ± s.d. of three biological repeats.


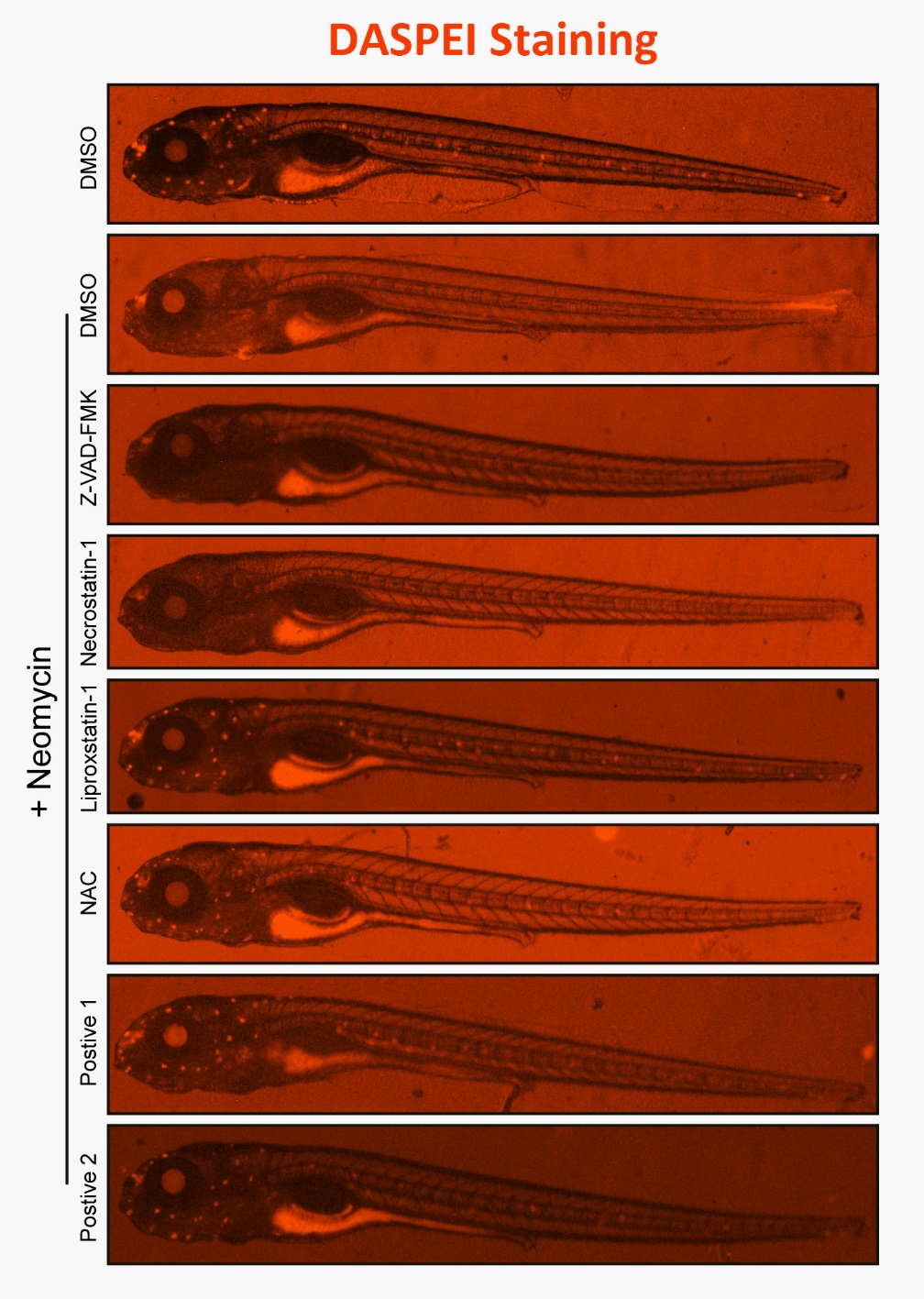


**Fig. S3. Visual screening for otoprotectants against neomycin-induced hair cell death in zebrafish larvae.** Larvae at 5 *dpf* were treated with a testing compound (10 µM) and neomycin (125 µM) for 2 h, stained with DASPEI then examined under a fluorescence microscope. At least 10 larvae were examined for each treatment and representative images are shown here.

**Table S1. List of alkaloid natural compounds used in the screen.**

| **Cat #** | **Compound Name** |
| --- | --- |
| T7056 | Dronedarone |
| T0131 | Cepharanthine |
| T3122 | (+)-Fangchinoline |
| T5S1103 | Isoliensinine |
| T2920 | Berbamine |
| T4614 | Ellipticine |
| T0690 | Quinine |
| T5S1099 | Liensinine |
| T2782L | Catharanthine tartrate |
| T5S2360 | Corydaline |
| T4S0795 | Berberrubine |
| T3054 | Daurisoline |
| T3369 | Nuciferine |
| T2874 | Tetrahydroberberine |
| T3246 | Tabersonine |
| T2809 | (+)-Thalrugosine |
| T5807 | Stylopine hydrochloride |
| T2S2215 | Crebanine |
| T8195 | Lotusine |
| T5810 | Tabersonine hydrochloride |
| T7786 | Tryptanthrin |
| T6S0119 | Dauricine |
| T4S2063 | Tetrahydrocoptisine |
| T5S1895 | Norisoboldine |
| T5S1097 | Neferine |
| T3S0807 | Berbamine |
| T8149 | Dobutamine (hydrochloride) |
| TN1091 | Dehydronuciferine |
| T5720 | Boldine |
| T8187 | Tetrahydroepiberberine |
| T0791 | Reserpine |
| TN1969 | N-(p-Coumaroyl) serotonin |
| TN2252 | Syrosingopine |
| TQ0296L | N-Nornuciferine hydrochloride(4846-19-9 free base) |
| T3S0057 | Dihydrochelerythrine |
| TN1032 | Geissoschizine methyl ether |
| T2714 | Rotundine |
| T2S1792 | Lycobetaine |
| T4S0794 | (R)-(+)-Corypalmine |
| T3152 | Harmol |
| T2832 | S-Isocorydine(+) |
| T4S2012 | Dihydro Sanguinarine |
| T2917 | Tetrahydropalmatine hydrochloride |
| T5S0814 | Berberine hydrogen sulphate |
| T8189 | Dihydroberberine |
| T5S2357 | Acetylcorynoline |
| T3S1457 | Palmatrubine |
| T3419 | Chelerythrine chloride |
| T4S0779 | D-tetrahydropalmatine |
| T8859 | Nitidine |
| T3933 | Jatrorrhizine |
| T2996 | Tetrandrine |
| T0461 | Berberine hydrochloride |
| T2793 | Tetrahydropalmatine |
| T3S1852 | Cephaeline hydrochloride |
| T4429 | Rauwolscine hydrochloride |
| T2781 | Sanguinarine |
| T3232 | Higenamine hydrochloride |
| T5S0056 | Coptisine chloride |
| T2868 | Evodiamine |
| T1668 | Vinblastine sulfate |
| TN1189 | 13-Methylberberine |
| T4S0800 | Demethyleneberberine |
| T8039 | Brucine sulfate heptahydrate |
| T5S2361 | Epiberberine |
| T8185 | Viroallosecurinine |
| T5760 | Sinoacutine |
| TN1142 | 8-Oxycoptisine |
| T1287 | Synephrine |
| T4036 | Solasodine |
| T7671 | Myosmine |
| T1286 | Vincamine |
| T8182 | Guan-fu base A |
| T5S0053 | Coptisine |
| T6213 | Vinorelbine tartrate |
| T1644 | Dopamine hydrochloride |
| T4S0051 | Coptisine sulfate |
| T5750 | Oxyberberine |
| T4592 | Tomatidine hydrochloride |
| T6S0657 | Isorhyncophylline |
| T6S0781 | Phellodendrine |
| T5800 | Allosecurinin |
| T3243 | Betaine |
| T3S1888 | Deltaline |
| T3S2340 | Usaramine |
| T3324 | Lycorine |
| T6S0659 | Rhynchophylline |
| T5S2102 | Leonurine |
| T5S0661 | Koumine |
| T3S1320 | Magnoflorine iodide |
| T8206 | Cycleanine |
| T2S2108 | Coixol |
| T8120 | N-Benzylstearamide |
| T6S1010 | Allomatrine |
| T4S0537 | Bullatine B |
| T5811 | Hyoscine hydrochloride |
| PDK0060 | Pseudotropine |
| T6S0084 | Tuberstemonine |
| T4963 | Higenamine |
| T7557 | Guvacine hydrochloride |
| T6569 | L-Mimosine |
| T8161 | Vicine |
| T6S1880 | Benzoylaconitine |
| T2803 | Monocrotaline |
| T4953 | Neotuberostemonine |
| T2142 | Yohimbine hydrochloride |
| T0086 | Galanthamine HBr |
| T6S1884 | Benzoylhypacoitine |
| T6377 | Aloperine |
| T3S0478 | Scopolamine |
| T7853 | Pachycarpine |
| T8016 | N-Benzylmethylamine |
| TN2239 | Strictosamide |
| PDK0014 | 3-Methylxanthine |
| T7975 | Rhodamine B |
| T2S1008 | Oxysophoridine |
| T8207 | Evolitrine |
| T0970 | Anisodamine |
| Fr16741 | Oxindole |
| T8298 | Vasicine hydrochloride |
| T3251 | Stachydrine hydrochloride |
| T3S2105 | N-Benzylpalmitamide |
| Fr13711 | Tropinone |
| T2986 | Jatrorrhizine hydrochloride |
| T5543 | Triacetonamine |
| T2811 | Harmine hydrochloride |
| TN1129 | Protostemotinine |
| T2726 | Sinomenine |
| T8307 | Hydroxy-​α-​sanshool |
| T5901 | 3-Hydroxy-2-methylpyridine |
| T5S0662 | Gelsemine |
| T7044 | Norepinephrine |
| TQ0192 | Senecionine |
| TN1925 | Methyl anthranilate |
| T5868 | Methyl 3-indolecarboxylate |
| T5S1889 | Yunaconitine |
| T7846 | Aegeline |
| T5693 | Methoxatin disodium salt |
| T3S1873 | Talatisamine |
| T0052L | Strychnine sulfate |
| T5S1882 | Napellonine |
| T10990 | Dehydrocorydaline chloride |
| T4S1321 | Magnoflorine chloride |
| T3158 | Harmane |
| T5S0106 | Peimisine |
| T0486 | Irinotecan hydrochloride trihydrate |
| T6S1885 | Benzoylmesaconine |
| T0647 | Tryptamine |
| TN3695 | Coniine hydrochloride |
| TN1899 | Lycoramine |
| T5769 | Stachydrine |
| T3901 | Solasonine |
| TN6712 | Yibeissine |
| T2S0112 | Yibeinoside A |
| T19792 | 1,3,7-Trimethyluric acid |
| TN1078 | Seneciphylline |
| T2850 | (+)-Bicuculline |
| T2890 | Hordenine |
| T20712 | Conessine |
| TL0001 | dencichine |
| T5S0658 | Corynoxine |
| T2S2335 | Dehydroevodiamine |
| T3S0629 | Delsoline |
| T7026 | KukoaMine B |
| T3S1319 | Magnoflorine |
| T4550 | Ajmaline |
| Fr16605 | Lupinine |
| T0130 | Physostigmine Salicylate |
| T1711 | Harmine |
| T3S1227 | Aristololactam I |
| T8287 | Sipeimine-3β-D-glucoside |
| T1591L | Cytidine |
| T0486L | Irinotecan hydrochloride |
| T12039 | Miglustat |
| T6981 | Nudifloric acid |
| T3S2100 | Securinine |
| T2725 | Scopolamine HBr |
| T6S0109 | Sipeimine |
| T3S1957 | 9-Aminocamptothecin |
| T6S0052 | Chelerythrine |
| T2797 | Harmaline |
| TN2003 | Obtucarbamate A |
| T2S0663 | Humantenmine |
| T3S0128 | Hydroprotopine |
| T3366 | Cephalotaxine |
| T1521 | Hydroxy camptothecine |
| T5805 | Norarecoline hydrochloride |
| T0479 | Cytisine |
| T4S1619 | Hyoscyamine sulfate hydrate |
| T5143 | Corynoxine hydrochloride(6877-32-3(free base)) |
| T8175 | Laudanosine |
| T2921 | Sinomenine hydrochloride |
| T6S0654 | Isocorynoxeine |
| T4S1725 | Galanthamine |
| TJS0312 | 7-Hydroxy-4-methyl-8-nitrocoumarin |
| T3S1729 | Dihydrolycorine |
| T2182 | Scopolamine HBr trihydrate |
| T3026 | (-)-Huperzine A |
| T5718 | L-Abrine |
| T5S0994 | N-Methylcytisine |
| T2181 | Scopolamine N-oxide HBr |
| T0019 | Betaine hydrochloride |
| T5853 | Rubitecan |
| T2887 | Trigonelline |
| T6S0033 | 2-Hydroxyadenosine |
| T5786 | Tetrahydropiperine |
| T5S0273 | Hypaphorine |
| TWS0704 | N-Methylnuciferine |
| TWP2911 | Thymidine |
| T2935 | Phenethylamine |
| T4S0536 | Bullatine A |
| T4S1869 | 12-Epinapelline |
| T0069 | Uracil |
| T5595 | Tropine |
| T1270 | Vincristine sulfate |
| T6S0630 | Hypaconitine |
| T3A2467 | Allocryptopine |
| T5814 | Aurantiamide |
| T4080 | Leonurine hydrochloride |
| T6S0627 | Mesaconitine |
| T3363 | Jervine |
| T2183 | Scopolamine butylbromide |
| T3339 | Sophoridine |
| T5S1891 | Acetylaconitine |
| T2947 | Indole-3-carbinol |
| T6S0107 | Peimine |
| T3S0631 | Fuziline |
| T5S0761 | Nitidine chloride |
| T5S1708 | Dendrobine |
| T8196 | Arborin |
| T6S2356 | (+)-Corynoline |
| T1062 | Capsaicin |
| T2S1720 | Huperzine B |
| T5S0802 | Palmatine |
| T0375 | Atropine |
| T4035 | Khasianine |
| T2S1200 | Sinapine |
| T5S0055 | Chelidonine |
| T0012 | Cinchonine |
| T5S0803 | Columbamine |
| T6S0105 | Peiminine |
| T1681 | Aminophylline |
| T5799 | Arecaidine hydrochloride |
| T3S1892 | Crassicauline A |
| T0167 | Vinpocetine |
| T0925 | Allantoin |
| T2870 | (+)-Matrine |
| T3S0970 | Thermopsine |
| T2198 | Arecoline HBr |
| T0437 | Inosine |
| T5061 | Lumichrome |
| T2993 | Cordycepin |
| T5765 | ranaconitine |
| T2718 | Palmatine chloride |
| T3030 | L-Sophoridine |
| T0804 | (+)-Pilocarpine hydrochloride |
| T6560 | Lappaconite HBr |
| T6S0655 | Corynoxeine |
| T2786 | Oxysophocarpine |
| T1362 | 6-Hydroxypurine |
| T7126 | Hydroquinine |
| T3S1967 | Abrine |
| T2149 | Eburnalritardo |
| T7938 | Quinidine |
| T1083 | Theophylline |
| T2773 | Vindoline |
| T7054 | N-Benzoyl-(2R,3S)-3-phenylisoserine |
| T0853 | Adenosine |
| T0962 | L-Hyoscyamine |
| T2754 | Oxymatrine |
| T2927 | Trigonelline hydrochloride |
| T2825 | Cyclopamine |
| T4952 | 10-Hydroxycampothecin |
| TN2085 | Piperlonguminine |
| T0792 | (-)-Sparteine sulfate pentahydrate |
| T5S1952 | 9-Methoxycamptothecin |
| T3002 | Piperine |
| T3364 | Veratramine |
| T4S0111 | Hupehenine |
| T2806 | Lappaconitine |
| T0934 | Nicotinamide |
| T2764 | 10-Hydroxycamptothecin |
| T2217 | Cephalomannine |
| T1156 | Palonosetron hydrochloride |
| T0186 | Docetaxel trihydrate |
| T5746 | Dictamnine |
| T2746 | Sophocarpine |
| T3380 | Homoharringtonine |
| T0190 | Vinorelbine |
| T1703 | SN38 |
| T4601 | 9-Methoxycanthin-6-one |
| T0320 | Colchicine |
| T1231 | Pilocarpine nitrate |
| T1174 | Topotecan hydrochloride |
| T1317 | Cinchonidine |
| T3S1955 | 7-Ethylcamptothecin |
| T0266 | Quinidine hydrochloride |
| T1334 | Hydroquinidine |
| TN1305 | 6-Ethoxydihydrosanguinarine |
| T8724 | 6-Methoxydihydrosanguinarine |
| TN6705 | Graveoline |
| T7939 | Fingolimod |
| T8892 | Emetine |
| T2774 | Lycorine chloride |
| T6947 | Piperlongumine |
| T4034 | Solamargine |
| T1123 | (S)-(+)-Camptothecin |
| T8286 | Harringtonine |
| T2972 | Rutaecarpine |
| T3786 | Tomatine |
| T0129 | Sanguinarine chloride |
| TN1012 | Febrifugine |
